# Metallosis following hip arthroplasty: two case reports

**DOI:** 10.1186/s13256-022-03336-4

**Published:** 2022-03-23

**Authors:** M. Mastel, A. Boisvert, R. Moore, F. Sutherland, J. Powell

**Affiliations:** 1 Division of Orthopedic Surgery, Department of Surgery, University of Saskatchewan, 107 Wiggins Road, Saskatoon, SK S7N 5E5 Canada; 2 Section of Orthopedic Surgery, Department of Surgery, University of Calgary, 2500 University Drive NW, Calgary, AB T2N 1N4 Canada; 3 Section of Vascular Surgery, Department of Surgery, University of Calgary, 2500 University Drive NW, Calgary, AB T2N 1N4 Canada; 4 Section of General Surgery, Department of Surgery, University of Calgary, 2500 University Drive NW, Calgary, AB T2N 1N4 Canada

**Keywords:** Pseudotumor, Metallosis, Total hip arthroplasty, Hip resurfacing, Femoral vessels, Vascular compression

## Abstract

**Background:**

There has been increasing recognition of local and systemic adverse events associated with the release of metal ions and nanoparticles from hip arthroplasty components. Adverse local tissue reactions to metal ion debris can include periprosthetic solid and cystic masses known as pseudotumors. These masses can result in pain, swelling, extensive destruction to surrounding hip soft-tissues, and compression syndromes on neurovascular, gastrointestinal, and genitourinary structures. As reports of pseudotumors requiring multidisciplinary excision are limited, we present two pseudotumor cases that were excised through a combined approach.

**Case presentations:**

The first case involves a 60-year-old Caucasian female with a large pseudotumor with intrapelvic and vascular involvement associated with a metal-on-polyethylene total hip arthroplasty, excised with contributions from general surgery, vascular surgery, and orthopedic surgery. Pseudotumor excision was followed by a revision total hip reconstruction in addition to an abductor mechanism reconstruction with tendo-Achilles allograft. The second case is that of a 64-year-old Caucasian female with a pseudotumor in close relationship to the femoral vessels following a metal-on-metal hip resurfacing, excised with a combination of vascular surgery and orthopedic surgery, with subsequent revision total hip reconstruction.

**Conclusions:**

There remains a lack of literature to support the extensiveness of pseudotumor excision required in complex cases with significant intrapelvic or vascular involvement. Given the potential for significant adverse effects of large masses, the authors’ preference is to involve a multidisciplinary team to achieve a more comprehensive excision while minimizing the risk of potential complications.

## Introduction

Total hip arthroplasty (THA) is a very common procedure to treat hip osteoarthritis, with over one million patients undergoing the procedure every year [1]. There has been increasing recognition of local and systemic adverse events associated with the release of metal ions and nanoparticles from arthroplasty components. This awareness initially developed following the introduction of large diameter metal-on-metal (MoM) THAs, and hip resurfacing devices with an MoM articulation [2, 3]. There are many potential advantages to MoM articulations, including their tolerance of high-impact loading, extremely low wear rates, and increased stability provided by the larger head size [2]. However, these components ultimately were found to have significantly higher revision rates up to three times those of contemporary hip implants with a resultant significant decline in their use [2–4]. A majority of these failures are attributed to the production of metal debris resulting in adverse local tissue reactions (ALTRs), a term used to describe a broad spectrum of pathological changes including extensive tissue necrosis, osteolysis, and large sterile hip effusions, as well as periprosthetic solid and cystic masses known as pseudotumors [1–3, 5, 6]. Although originally felt to be the result of the MoM articulation itself, there has been increasingly implication of these nonmalignant and noninfectious masses and ALTRs in other THA articulations, including metal-on-polyethylene (MoP) and ceramic-on-polyethylene (CoP) bearings, likely the result of metal ion release from the modular junctions [1, 2, 5, 7–10]. Although patients may be asymptomatic, pseudotumors may result in pain, swelling, extensive destruction to surrounding hip soft tissues, and compression syndromes on neurovascular, gastrointestinal, and genitourinary structures [1, 2, 5, 6, 11–15]. As reports of extensile pseudotumors requiring multidisciplinary excision are limited, we present two cases of pseudotumor following hip arthroplasties that involved vascular and intrapelvic structures that were excised through a combination approach.

## Case 1

**Fig. 1:**
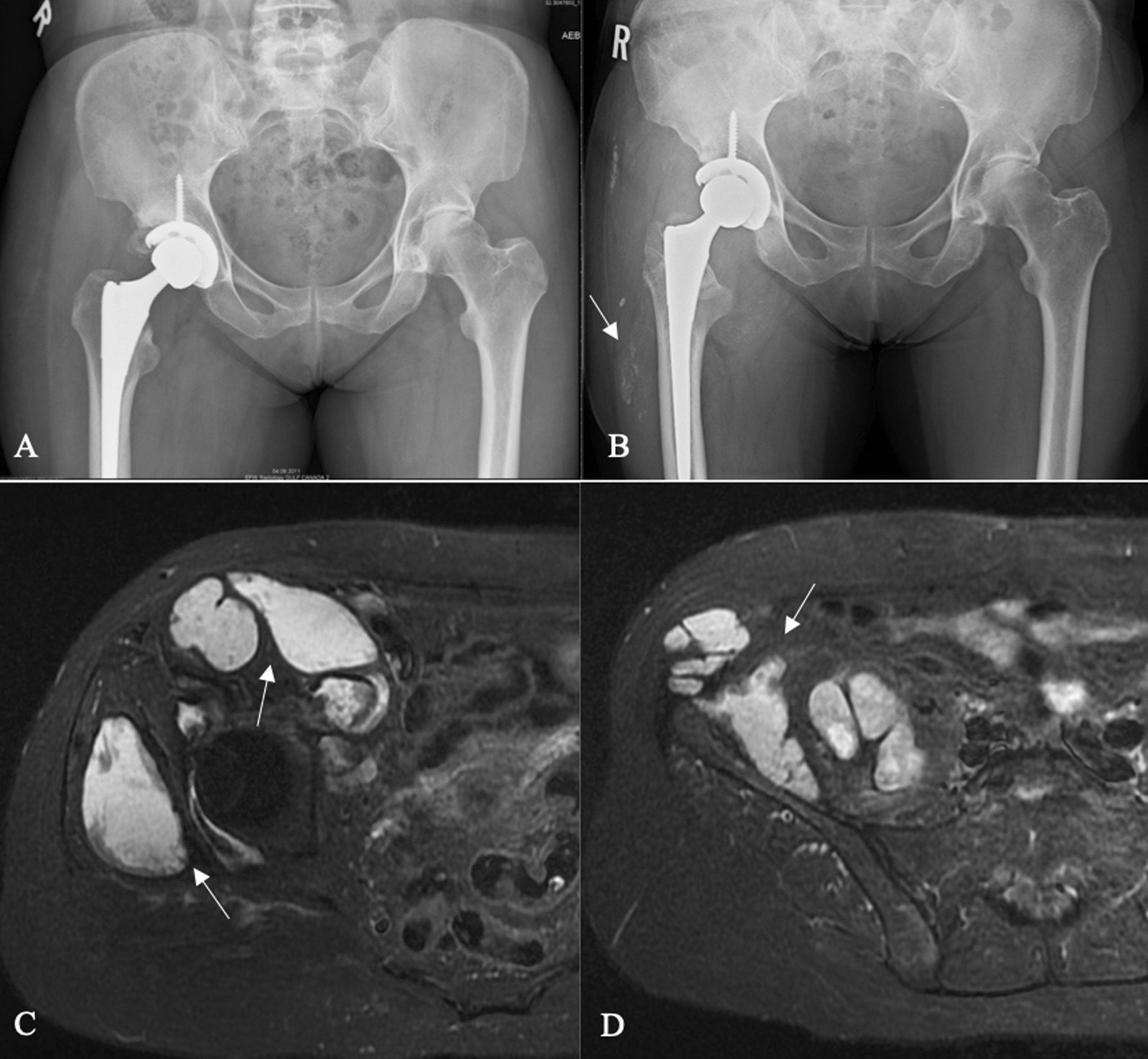
Select preoperative images: **A** anteroposterior (AP) pelvis radiograph following index right THA; **B** AP pelvis radiograph prior to revision demonstrating soft-tissue shadowing and mineralization surrounding the right hip and proximal femur (arrow); **C**, **D** axial MRI slices revealing significant pseudotumor extension (arrows)

**Fig. 2:**
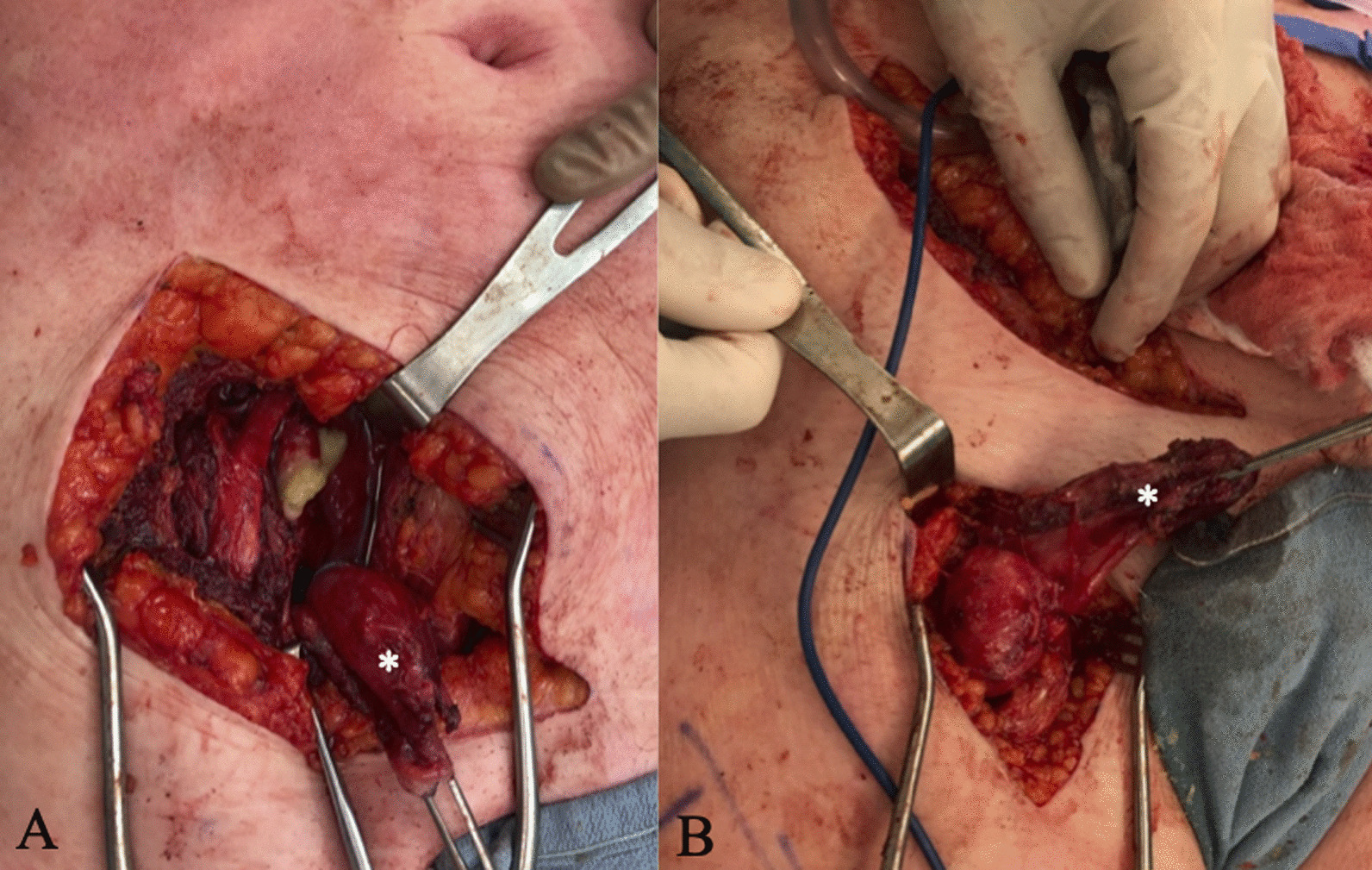
Intraoperative images demonstrating **A** anterior intrapelvic pseudotumor excision; **B** second incision in femoral compartment (asterisks indicate pseudotumor)

**Fig. 3:**
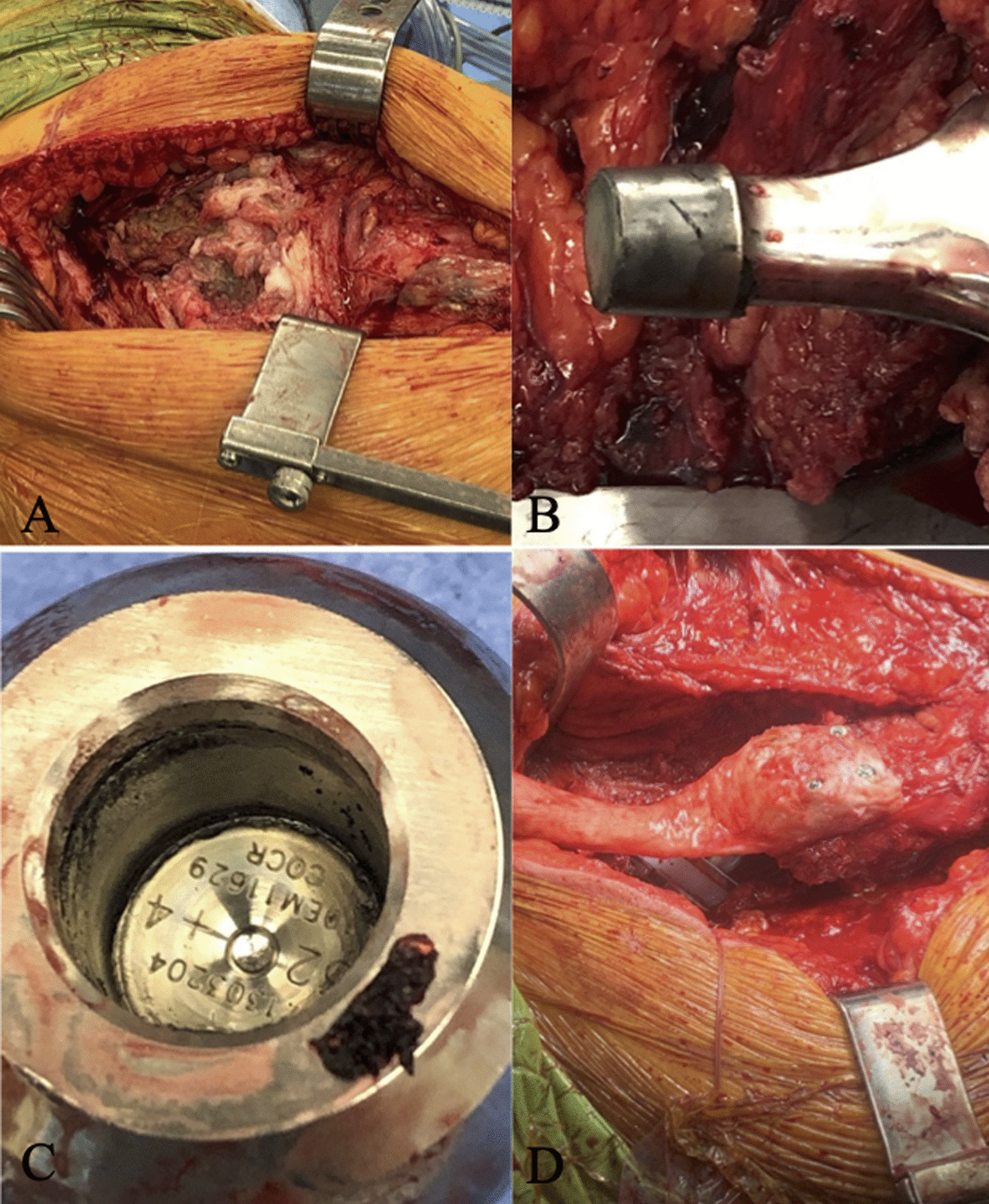
**A** Significant lateral pseudotumor with near-complete destruction of abductor musculature; **B**, **C** trunnionosis with wear and corrosion products involving the femoral neck trunnion and bore of the femoral head; **D** abductor mechanism reconstruction with tendo-Achilles allograft prior to proximal tensioning

**Fig. 4:**
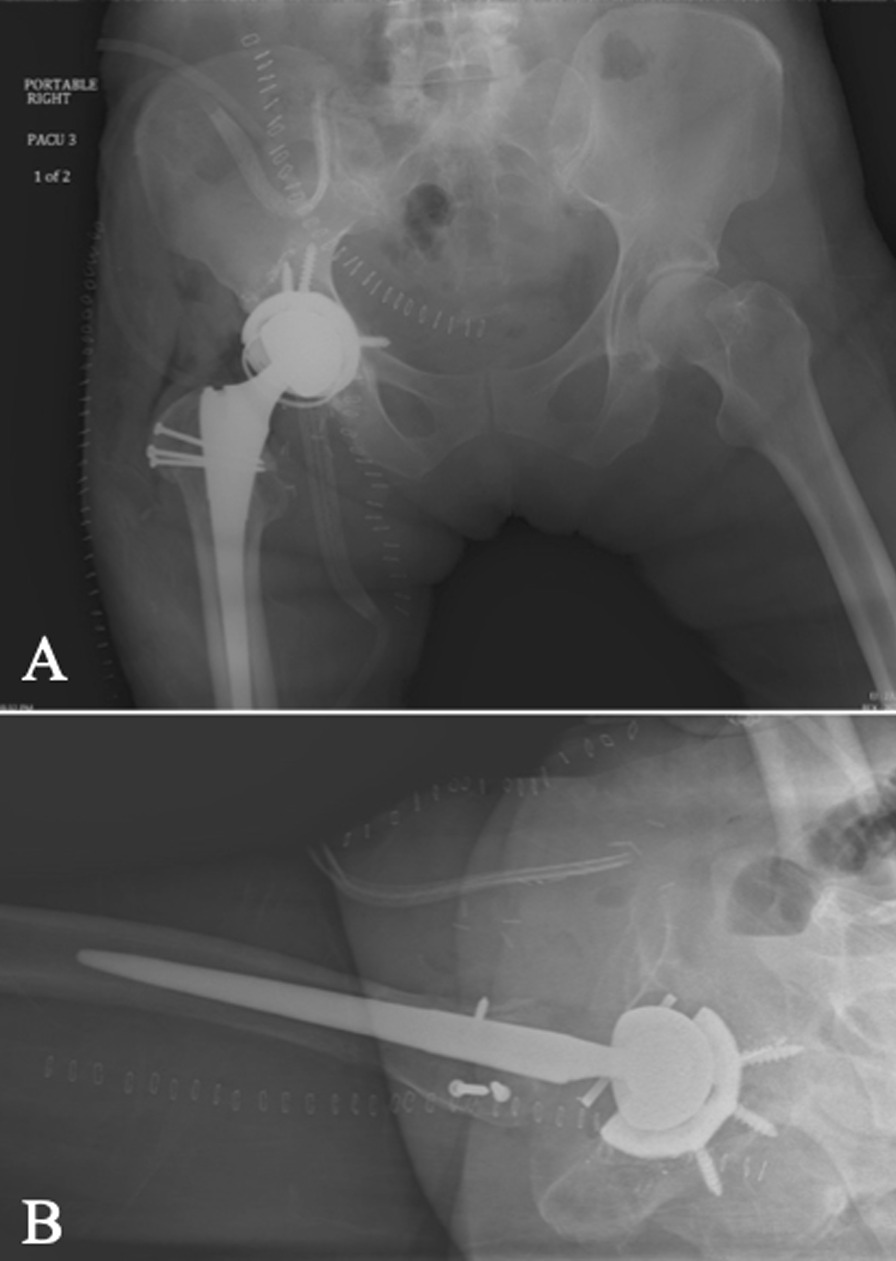
Immediate anteroposterior (**A**) and lateral (**B**) postoperative radiographs of right revision total hip arthroplasty following excision of extensive pseudotumor through multiple approaches

A 60-year-old Caucasian female had undergone right primary THA for end-stage osteoarthritis. Components utilized include a Smith and Nephew R3 50 mm acetabular shell, R3 cross-linked polyethylene liner, size 11 Synergy femoral stem, and 32 mm diameter + 4 mm neck length cobalt-chrome femoral head. The arthroplasty was performing well until a dislocation 8 years following implantation. She ultimately re-presented to the initial orthopedic surgeon (JP) 9 years, 7 months following the index surgery after sustaining a second dislocation. At this point the patient was essentially asymptomatic with no pain, and was still functioning well apart from hip instability. Examination revealed a fluctuant, nontender collection anterior to the hip joint, along with a painless range of motion (ROM) including 90° flexion, 30° internal rotation (IR), and 30° external rotation (ER). Radiographs suggested radiolucency in the greater trochanter, and soft tissue shadowing and mineralization anterior to the joint, and along the lateral proximal femur (Fig. 1B). Subsequent metal artifact reduction sequence (MARS) magnetic resonance imaging (MRI) revealed complex multiloculated fluid collections suggestive of extensive pseudotumor originating from the right total hip arthroplasty, extending laterally and superiorly into the hip abductors, anteriorly and superiorly along psoas musculature, and along the inner table of the pelvis to the level of the iliac crest. Inferior extension continued to the deep aspect of the hamstring tendon origins (Fig. 1C, D). The pseudotumor was in close contact with the external iliac artery and vein and was displacing the femoral nerve. Laboratory investigations including a C-reactive protein (CRP) level of 2.1 mg/L and erythrocyte sedimentation rate (ESR) of 12 mm/hour suggested a low likelihood of infection. Assessment of whole blood metal ion levels revealed a cobalt level of 57.5 nmol/L (approximately 3.39 ppb) and chromium level of 11.2 nmol/L. Six weeks following confirmation of the associated pseudotumor, the patient underwent a near-complete excision of the intra- and extrapelvic pseudotumor followed by revision total hip arthroplasty and abductor mechanism reconstruction through a multidisciplinary team approach. With the patient supine, general surgeon (FS) performed a laparotomy in the right lower abdominal quadrant (Fig. 2). In conjunction with a vascular surgeon (RM), the pseudotumor was found to extend from the retroperitoneal space displacing the iliac vessels and femoral nerve. There were numerous extensions into the psoas muscle, and a small amount of pseudotumor tissue was left in this area to minimize morbidity. Extension posteriorly invading the iliac crest also resulted in some retained tissue. The pseudotumor extended inferiorly beneath the inguinal ligament into the anterior femoral compartment and was excised through a second incision in the groin longitudinally over the femoral artery (Fig. 2). Following anterior pseudotumor excision, the patient was repositioned in the lateral decubitus position, and through the previous THA incision a posterolateral approach was performed. Pseudotumor was encountered on incising the deep fascia. The abductors were nearly completely destroyed by pseudotumor (Fig. 3A), which was carefully excised. Trunnion wear was noted with black deposits on the femoral neck (Fig. 3B) and in the bore of the femoral head (Fig. 3C). The wear debris was cleaned off the neck of the femoral component, and the anteversion and fixation of the stem were confirmed to be appropriate before retention. Explantation of the fixed acetabular component revealed mild posterior bone loss, and press-fit fixation of a Smith and Nephew 52 mm R3 socket was augmented with two fixation screws superiorly and one posteroinferiorly (Fig. 4). In view of the absent abductors, a constrained liner was utilized with a 32 mm diameter + 4 mm neck length Oxinium head. Abductor reconstruction with tendo-Achilles allograft was performed with the os calcis contoured and secured to the greater trochanter and tendon sutured to the gluteus medius fascia with the hip abducted (Fig. 3D). The patient was instructed on toe-touch weight-bearing for 6 weeks with an abductor orthosis. The histopathological report revealed a final diagnosis of “fibroconnective tissue with granulomatous and giant cell reaction, with foreign bodies and calcifications, negative for malignancy.” Final culture results all returned negative. At 6 weeks follow-up, abductor strength was 4/5 and the patient was recovering well.

## Case 2

**Fig. 5:**
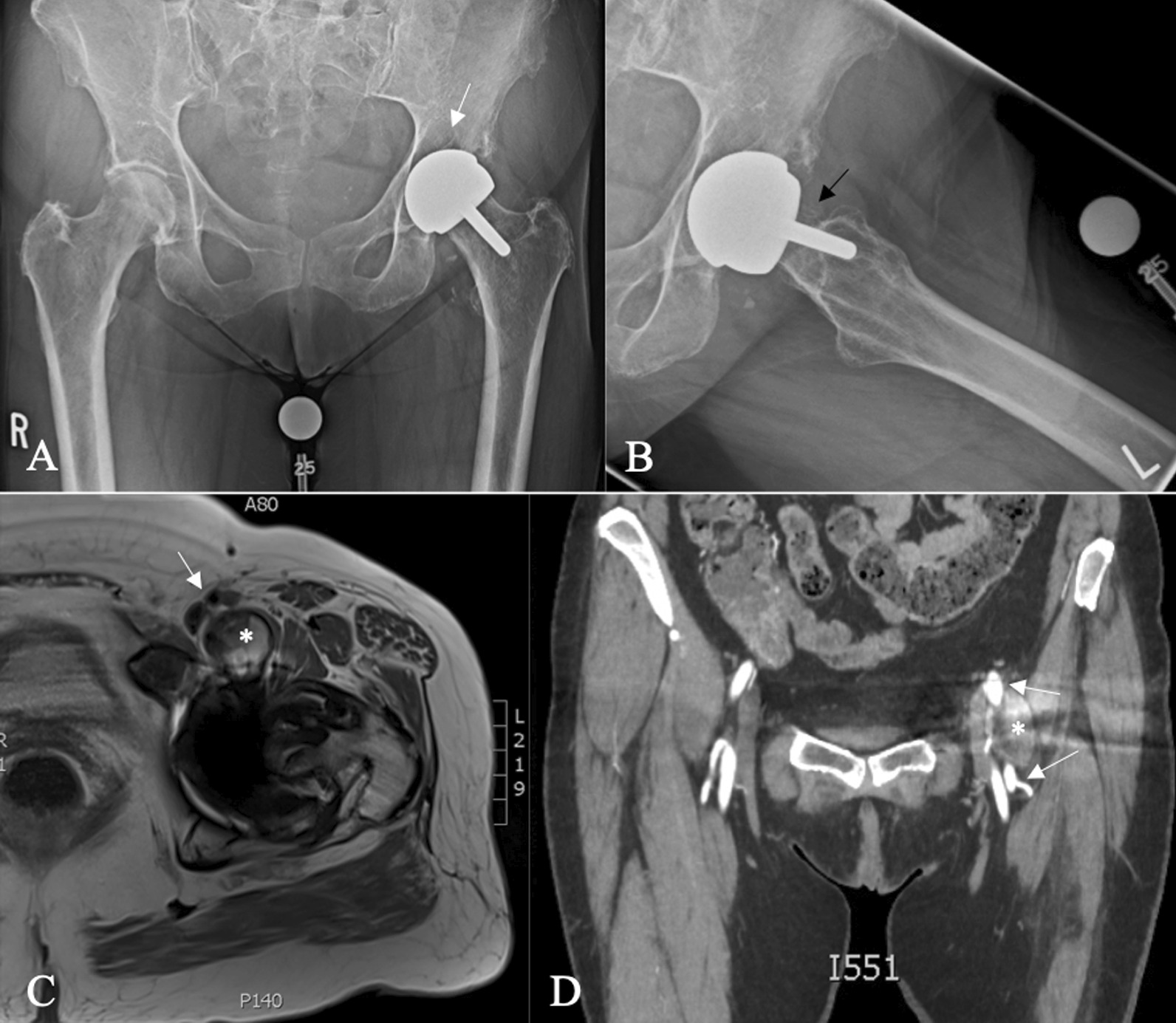
Select preoperative images including **A**, **B** AP pelvis and lateral left hip radiographs demonstrating acetabular osteolysis (white arrow) and erosion of the anterior femoral neck (black arrow); **C** axial MRI slice with anterior pseudotumor (asterisk) abutting the posterior aspect of the femoral neurovascular bundle (arrow); **D** coronal CT angiogram slice again illustrating the close proximity of the pseudotumor (asterisk) to the left femoral vessels (arrow)

**Fig. 6:**
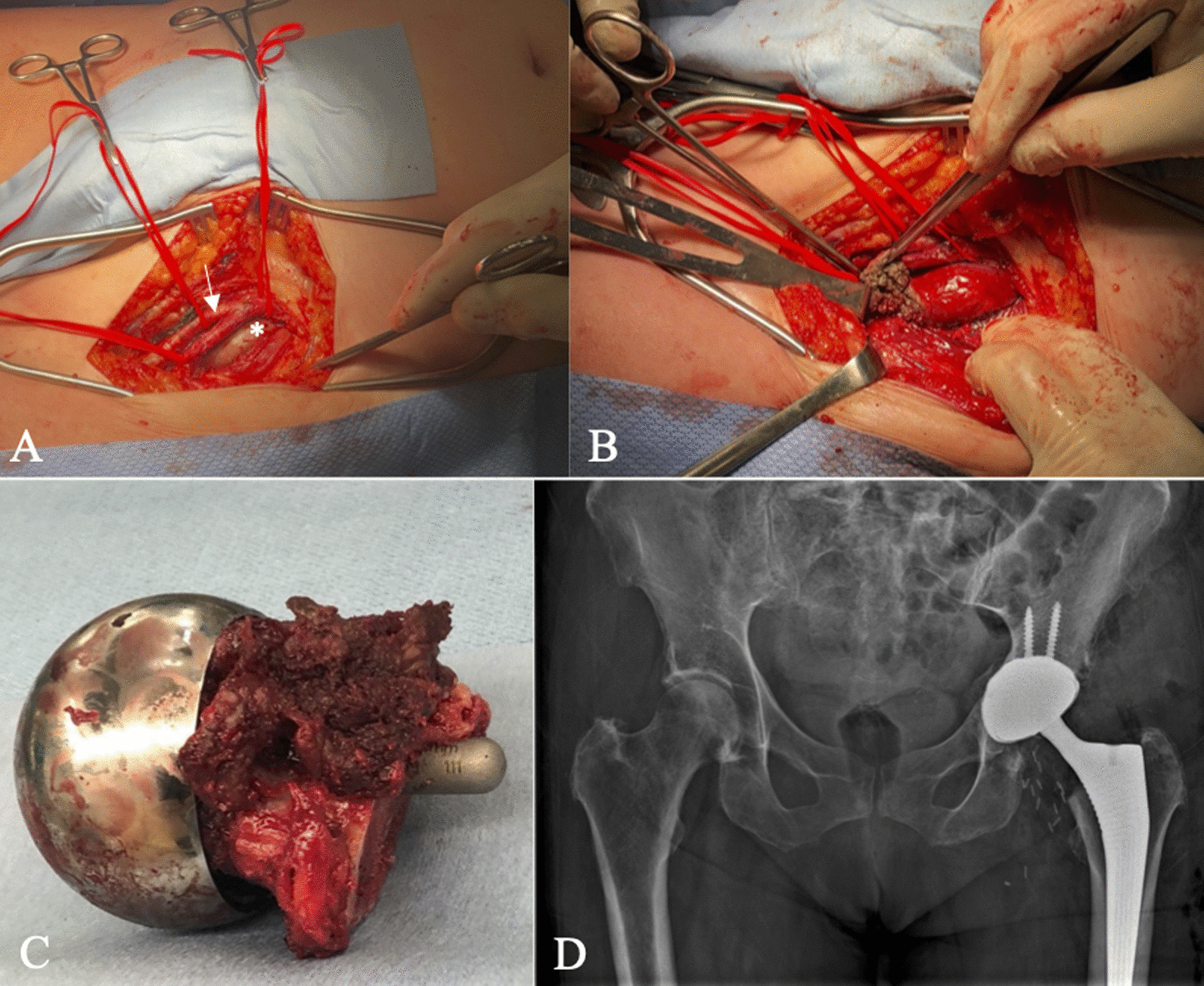
Intraoperative images and immediate postoperative radiograph demonstrating **A** anterior pseudotumor (asterisk) dissection from the posterior aspect of the superficial femoral artery (arrow), profundal femoris, and femoral nerve; **B** the pseudotumor was ultimately taken out piecemeal to excise it safely; **C** pseudotumor was adherent to the anterior femoral neck and resulted in significant bone erosion; **D** immediate postoperative AP pelvis X-ray of the conversion to total hip arthroplasty

A 64-year-old Caucasian female referred to orthopedic surgery (JP) for urgent assessment of elevated metal ion levels and left hip pseudotumor. She had undergone a left Smith and Nephew Birmingham hip resurfacing (BHR) for end-stage osteoarthritis 15 years prior, with a hip arthroscopy having been performed prior to that. Components utilized included a 52 mm BHR acetabular cup and 46 mm BHR resurfacing head. The arthroplasty was performing well until 5 years prior when the patient noticed intermittent bothersome squeaking that since resolved, as well as progressive anterior hip discomfort described as pressure sensation with sharp positional pains. Walking duration was limited to two to five blocks without mobility aids. Medical history includes depression, hypertension, and 40 pack-year smoking history. A review of systems also revealed progressively worsening vision, and intermittent paresthesia in her hands bilaterally. Examination revealed fullness anterior to the affected hip joint along with increased prominence of local superficial veins. ROM was 90° flexion, 0° IR with discomfort, 30° ER. Abductor strength was 5/5. Distal pulses remained symmetric, and neurological function was preserved. Radiographs suggested acetabular osteolysis and significant erosion of the anterior femoral neck (Fig. 5). MARS MRI revealed complex multiloculated collections suggestive of a pseudotumor originating from the left hip resurfacing, extending anterior in close proximity to the femoral neurovascular bundle (Fig. 5). The gluteus medius and minimus were intact. Subsequent computed tomography (CT) angiogram revealed medial displacement of the femoral vasculature with preserved distal circulation status (Fig. 5). Laboratory investigations including a CRP level of 1.9 mg/L and ESR of 10 mm/hour, suggesting a low likelihood of infection. Whole blood metal ion levels from 1 year prior revealed a concerning cobalt level of 1225.8 nmol/L (approximately 72 ppb) and chromium level of 389 nmol/L. Two weeks following initial consultation with orthopedic surgery, the patient underwent excision of the extrapelvic pseudotumor followed by conversion to total hip arthroplasty through a multidisciplinary team approach. With the patient supine, vascular surgeon (RM) performed an anterior dissection through a longitudinal incision. There was gross adherence of the pseudotumor to the back wall of the common femoral and femoral bifurcation (Fig. 6) that required sharp dissection and repair of the profundal femoris with a profundoplasty after removal of the tumor from the wall of the vessel itself. Superiorly, the tumor extended up under the inguinal ligament, and aggressive retraction was required to allow for complete mobilization of the lesion along the lymphatic chain; a complete retroperitoneal lymph node dissection was carried up to the level of the distal external iliac artery into the pelvis. Distally, it tracked to the anterior joint capsule. The lesion was removed in its entirety with the exception of the attachments. Following anterior pseudotumor excision, the patient was repositioned in the lateral decubitus position, and through the previous lateral incision a posterolateral approach was performed. The abductors were in excellent condition. Pseudotumor was encountered upon posterior capsulotomy and was adherent to inner capsule and the anterior femoral neck. Along with complete excision of pseudotumor the BHR components were explanted and a revision THA performed. Large contained defects were filled with cancellous allograft bone. Final components include an uncemented 54 mm Smith and Nephew R3 socket that was augmented with two fixation screws superiorly. A Smith and Nephew OR3O Oxinium dual mobility articulation was used with a size 6 uncemented standard offset Polar stem (Fig. 6). The histopathological report revealed a final diagnosis of “joint debris with associated histiocytic reaction, consistent with prosthesis-associated changes.” Final culture results all returned negative.

## Discussion

There are a variety of metal ions continually released from all hip prosthesis. However, abnormal wear or corrosion through numerous mechanisms can result in excessive metal ion generation [16]. Cobalt-chromium (CoCr) is the most common alloy used in articulating surfaces of hip implants and is composed of primarily cobalt, followed by chromium, molybdenum, and a small portion of other trace elements such as vanadium, manganese, silicon, iron, nickel, and carbon [1]. As a result, cobalt is the most commonly implicated and discussed metal ion in hip arthroplasty failure. In MoM arthroplasties, tribocorrosion at the articulating surfaces is felt to be the primary generator of metal ion release [1], while fretting corrosion and mechanically assisted crevice corrosion (MACC) have been demonstrated to be a particular mechanism of metal ion release at trunnion junctions and secondary modular interfaces in MoP and CoP components [1, 7, 8]. The pathophysiology of pseudotumor formation is complex and not completely understood, and has been discussed more extensively elsewhere [1, 2, 7].

The prevalence of asymptomatic pseudotumor related to MoM hip arthroplasties may be as high as 78%, with the revision rate for symptomatic pseudotumor ranging from 1.7% to 5.6% [17]. The prevalence in MoP articulations may be higher than previously believed, with one study demonstrating on MARS MRI a prevalence of 43% in MoM THA, 28% in MoM resurfacings, and, surprisingly, 41% in MoP THA [18]. However, symptomatic rates are significantly lower, with revision for ALTR in non-MoM bearings estimated to be 0.77% of all revisions in the United Kingdom National Joint Registry [7]. Unfortunately, the lack of recognition of this clinical problem may lead to under-reporting in joint registries, and one single-institution study of 2,409 primary MoP THAs revealed a higher revision rate of 0.5% for symptomatic pseudotumor at a mean follow-up of 7 years [8]. The presence of a pseudotumor in our first case report reinforces the potential significance of trunnionosis and the need for awareness of this mode of failure in MoP THAs, in addition to the routine surveillance recommended for MoM articulations.

Along with ALTRs, there has been increasing concern of the systemic effects of elevated metal ion levels, in particular cobalt and chromium. In addition to potential cobalt-induced myocardial toxicity with previous fatal reports, systemic effects reported include toxicity to the auditory, ocular, central, and peripheral nervous systems; thyrotoxicity; hematologic effects including polycythemia and changes to cell-mediated immunity; hepatotoxicity; carcinogenicity; teratogenicity; gastrointestinal symptoms; and dermatitis [7, 16]. Unfortunately, the association between metal ion levels and the resultant pathological effects are also complex and not well understood [16, 19]. In one systematic review of systemic cobalt toxicity with MoM THAs, the average serum cobalt level was 35 μg/L with a range of 14–288 μg/L [19]. The patient in the second case we present in this report had a cobalt ion level of 72 μg/L (1 μg/L = approximately 1 ppb) [19], and further evaluation by internal medicine is pending to assess potential systemic effects. However, it was noted that the patient did present with progressively worsening vision, bilateral hand paresthesias, and depression, which are all potential yet relatively nonspecific symptoms of toxicity.

Revision THA aims to decrease the excessive production of metal ions, and is often the definitive management of patients with symptomatic ALTRs, patients with pseudotumors in the presence of significantly elevated metal ion levels, or those with systemic symptoms of toxicity. Pseudotumor accessible through the revision hip approach is always excised, appropriate bearing surfaces are utilized to minimize future metal ion insult, and instability risk is addressed with increased levels of constraint and potentially reconstruction of the abductor mechanism if necessary.

Unfortunately, significant extension, including into the pelvis, can occasionally occur with large lesions, and there remain no well-defined criteria to guide the required extent of excision [5, 12–14]. Although patients with pseudotumors may be asymptomatic, there have been reports of these intrapelvic lesions resulting in abdominal pain resulting in admission under a general surgery service, gynecologic issues including uterovaginal prolapses with initial presentation to a gynecology service, urinary retention with hydronephrosis, compression of the external iliac vasculature or femoral ﻿vasculature from a mass extending anteriorly along the iliopsoas tendon, and femoral nerve palsy [5, 6, 11–15]. Patients with a femoral vein compression syndrome may present with only unilateral leg swelling with preserved distal pulses [6, 14, 15]. Given the potentially significant multisystem effects caused by large pseudotumor extension, the senior orthopedic surgeon (JP) prefers a multidisciplinary assessment including general surgery for any intrapelvic pseudotumor extension, and vascular surgery for any pseudotumor encroaching on major vascular structures. Similarly, the appropriate subspecialty surgical service should be involved during the workup of any extensile lesion encroaching on other vital local anatomy such as genitourinary structures. Internal medicine services should be involved in the evaluation and management of systemic effects of metallosis, and appropriate subspecialty medical services must be consulted when indicated. This may include ophthalmology for resultant ocular issues, otolaryngology for auditory effects, neurology for central or peripheral nervous system dysfunction, endocrinology for thyrotoxicity, hematology for resultant polycythemia or cell-mediated immunity changes, gastroenterology for gastrointestinal systems or hepatotoxicity, dermatology for dermatitis, and psychology for mental health changes as a result of elevated metal ion levels. Likewise, this multidisciplinary approach is utilized during surgical excision when vital anatomic structures are involved. It is felt that this may help reduce the occurrence of major complications that are described to occur in up to 50% of revision THAs for pseudotumor, and is an approach also recommended by numerous authors [5].

## Conclusion

Metal ion debris has been an increasingly recognized problem following hip arthroplasties of all bearing surfaces, with the potential to induce adverse local tissue reactions as well as rarely systemic toxicity. There remains a lack of literature to support the extensiveness of pseudotumor excision required in complex cases with significant intrapelvic or vascular involvement. Given the potential for significant adverse effects of large masses, the author’s preference is to involve a multidisciplinary team to achieve a more comprehensive excision while minimizing the risk of potential complications.

## Data Availability

Not applicable.
